# Structural flexibility of the periplasmic protein, FlgA, regulates flagellar P-ring assembly in *Salmonella enterica*

**DOI:** 10.1038/srep27399

**Published:** 2016-06-07

**Authors:** Hideyuki Matsunami, Young-Ho Yoon, Vladimir A. Meshcheryakov, Keiichi Namba, Fadel A. Samatey

**Affiliations:** 1 Trans-Membrane Trafficking Unit, Okinawa Institute of Science and Technology Graduate University, 1919-1 Tancha, Onna, Kunigami, Okinawa 904-0495, Japan; 2 Dynamic NanoM achine Project, International Cooperative Research Project, Japan Science and Technology Agency, 1-3 Yamadaoka, Suita, Osaka 565-0871, Japan; 3 Graduate School of Frontier Biosciences, Osaka University, 1-3 Yamadaoka, Suita, Osaka 565-0871, Japan; 4 Riken Quantitative Biology Center, 1-3 Yamadaoka, Suita, Osaka 565-0871, Japan

## Abstract

A periplasmic flagellar chaperone protein, FlgA, is required for P-ring assembly in bacterial flagella of taxa such as *Salmonella enterica* or *Escherichia coli*. The mechanism of chaperone-mediated P-ring formation is poorly understood. Here we present the open and closed crystal structures of FlgA from *Salmonella enterica* serovar Typhimurium, grown under different crystallization conditions. An intramolecular disulfide cross-linked form of FlgA caused a dominant negative effect on motility of the wild-type strain. Pull-down experiments support a specific protein-protein interaction between FlgI, the P-ring component protein, and the C-terminal domain of FlgA. Surface plasmon resonance and limited-proteolysis indicate that flexibility of the domain is reduced in the covalently closed form. These results show that the structural flexibility of the C-terminal domain of FlgA, which is related to the structural difference between the two crystal forms, is intrinsically associated with its molecular chaperone function in P-ring assembly.

The bacterial flagellum is a macromolecular assembly composed of about 30 different proteins with copy numbers ranging from several to tens of thousands^1^,^2^. *Salmonella enterica* serovar Typhimurium (*S. enterica*) is a motile, Gram-negative bacterium with peritrichous flagella that have been studied intensively by genetic, biochemical, and structural approaches. The bacterial flagellum consists of three major substructures: the basal body, the hook, and the filament. The basal body contains a rotary motor, composed of the MS-ring, the LP-ring, and the drive shaft, called the rod, which traverses the inner- and outer membranes of Gram-negative cells. Connected to the rod is the hook. It transmits motor torque to the filament, which forms a long helical coil that functions as a “propeller” outside the cell. The LP-ring is a molecular bushing that stabilizes high-speed rotation of the flagellum. It is a chemically stable substructure composed of about 26 copies each of FlgH and FlgI, associated with the bacterial outer membrane and peptidoglycan (PG) in the cell wall, respectively^3^,^4^. FlgH and FlgI, which comprise the L- and P- rings, respectively, are synthesized in the bacterial cytoplasm and exported to the periplasm by the Sec-dependent pathway^5^. Except for the bacterial Phylum Firmicutes, for which FlgH and FlgI are not necessary, due to the thick PG layer that holds the rod, the LP-ring is required by most Gram-negative bacteria to form functional flagella^6^. The L- and P-rings are anchored to the lipopolysaccharide layer of the outer membrane and the PG periplasmic layer^3^, respectively. After secretion into the periplasm by the Sec-dependent pathway, FlgH is subjected to the outer membrane sorting system *Lol*^7^,^8^, because it has a canonical cysteine residue that is modified with a lipid moiety for outer-membrane localization^9^.

In the cytoplasm, specific additional chaperones control flagellar assembly^10^,^11^,^12^. FlgA is a periplasmic flagellar protein that chaperones P-ring formation^5^,^13^,^14^. FlgA possesses a typical signal sequence at its N-terminus, recognized by the Sec-dependent pathway, suggesting that FlgA functions as a P-ring assembly chaperone in the periplasm^15^. Direct evidence of FlgA binding to FlgI was previously demonstrated by genetic and biochemical analyses^16^. P-ring formation is a key step enabling the bacterial flagellum to pass through the outer membrane^17^. L-ring formation depends on a pre-formed P-ring, without which L-ring assembly is severely impaired^13^. To gain insight into the regulatory mechanism of P-ring assembly by FlgA, two different atomic structures of FlgA from *S. enterica*, the open and closed forms were solved. The structures of these distinct forms reveal structural flexibility that is essential for FlgA function and P-ring formation. Our experiments on the non-flagellated strain of *S. enterica* SJW1446 show in greater detail, how flexibility of FlgA enables its chaperone function during P-ring assembly.

## Results

### Open and Closed Conformations of the Chaperone Protein, FlgA

**Figure 1:**
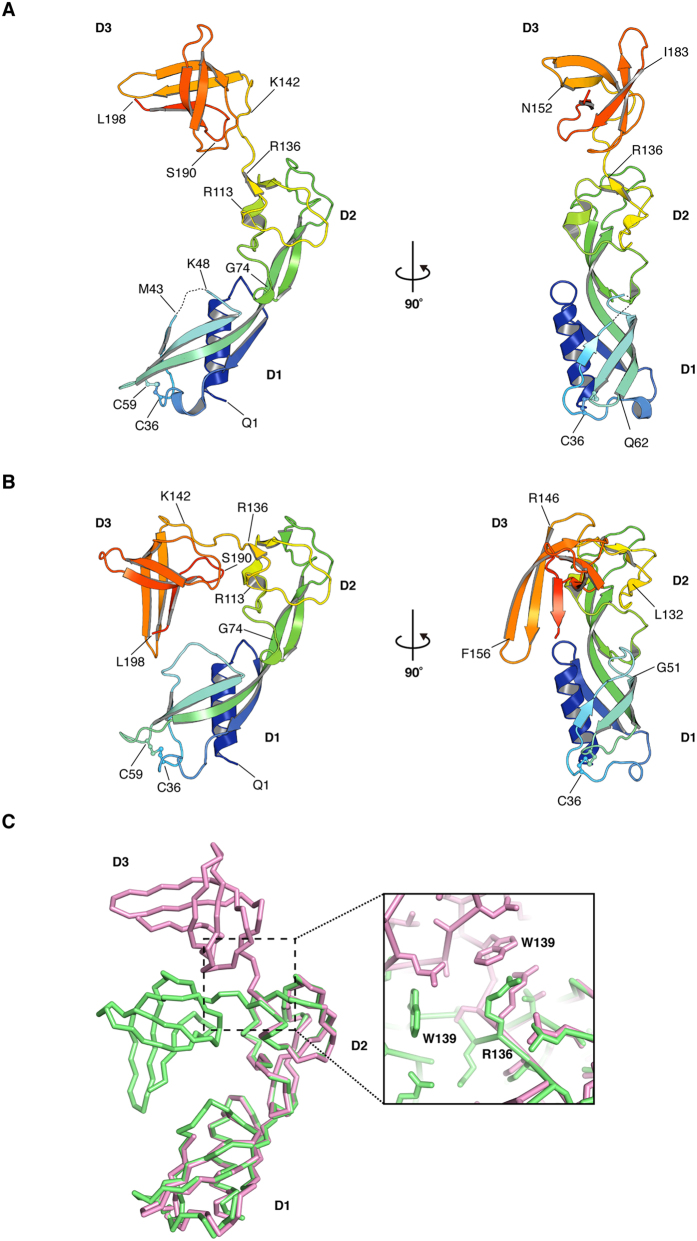
Overall structure of *S. enterica* FlgA. The chain is color-coded from blue to red from the N- to the C-terminus. Residues are shown as one-letter codes with numbering. The open structure of FlgA with the main secondary structure annotated with two rotations by 180° difference (**A**) and the closed structure of FlgA (**B**). Figures were prepared with PyMOL (The PyMOL Molecular Graphic System, Schrödinger, LLC. http://www.pymol.org.). (**C**) Comparison of FlgA structures in *ribbon*. The open form in *magenta* and the closed form in *lime* are aligned relative to their D2 domains. A close-up view of residues around R136 is also shown.

*S. enterica* FlgA was expressed as a C-terminal, hexa-histidine-tag-fused precursor and was purified from the periplasm of *E. coli* cells. Purification, crystallization, and diffraction data collection have been described previously^18^. We solved two crystallographic structures of FlgA. Atomic models were built from amino acid residues Q1 through L198 (numbering corresponds to the mature protein) for the open and closed forms (Fig. 1A,B) and refined at resolutions of 1.95 Å and 2.3 Å, respectively (Table 1). For the open form, a segment from T44 thorough A47 was not traced in the final model due to the poor quality of the electron density map in this region.

FlgA can be divided into three domains, denominated as D1 (residues 1–74), D2 (residues 75–142), and D3 (residues 143–198). The N-terminus begins with an amphipathic α-helix (α1), followed by a four-stranded, anti-parallel β-sheet. The first three β-strands, β1, β2, and β3, are within D1. The long, fourth β-strand, β4, stretches from D1 into D2. Domain D2 is composed of four short, anti-parallel β-strands linked by loops. Domain D3 is predominantly made of a five-stranded β-sheet (β8, β9, β10, β11, β12) that forms, a short β-barrel (Supplementary Fig. S1). The first four strands are anti-parallel, while the fifth C-terminal strand is parallel to the fourth. FlgA has two cysteine residues (C36 and C59) that form a disulfide bridge in both crystal structures and the side chain and the bond are visible in a ball-and-stick representation (Fig. 1A,B). These cysteines are not conserved throughout the FlgA family (Supplementary Fig. S1) and we found that the disruption of this disulfide bond does not affect motility of SJW1446 (Supplementary Fig. S2).

While the structures of corresponding domains are similar in the open and closed forms, the D1 domains of the two forms align with an RMSD of 0.64 Å, while the D2 and D3 domains align with RMSDs of 0.35 Å and 0.33 Å, respectively (Fig. 1C). The conformation of FlgA is different in the two forms (Supplementary Movie S1). In the closed form, D3 is closer to D1 and forms a compact structure. This occurs largely because of a bend of about 70° around the Cα atom of R136 (Fig. 1C).

### Comparison with the FlgA of a thermophile

**Figure 2:**
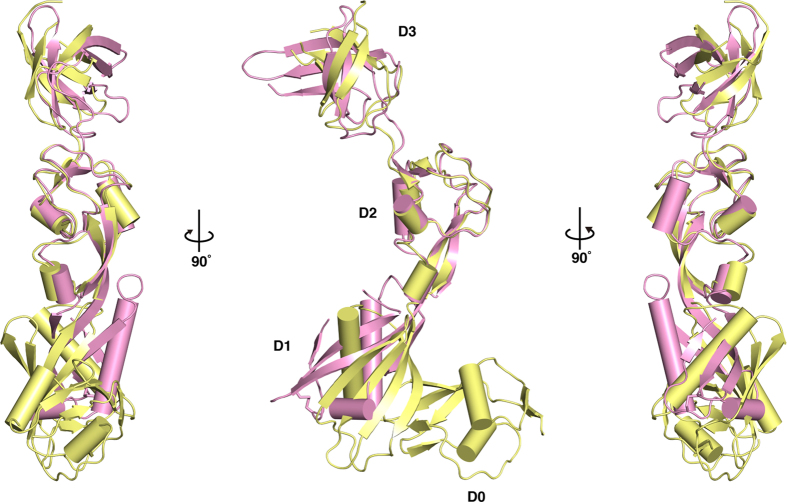
Structural comparison of FlgA proteins from *S. enterica* and *T. maritima*. Overall structures of *S. enterica* (PDB-id: 3TEE, *magenta*) and *T. maritima* (PDB-id: 3FRN, *paleyellow*) are shown as ribbon representations with cylindrical helices by superimposing the D2 domains.

The crystal structure of *Thermotoga maritima* FlgA, (*T. maritima*) containing residues 17–283 (out of 286), has been deposited in PDB by a structural genomics consortium. The structure contains four domains: D0 to D3. The N-terminal domain D0, which does not exist in *S. enterica*, is composed of residues 17–84. There is a slight diversity in the length of these terminal regions among FlgA proteins (Supplementary Fig. S1). Except for the D0 domain, FlgA from both *S. enterica* and *T. maritima* share similar domain organization. The overall structures of both FlgAs superimpose with a root-mean-square deviation (RMSD) of 2.7 Å^19^. The domains common to both FlgAs superimpose with RMSDs of 2.8, 1.2 and 1.3 Å, respectively^19^. FlgA from *T. maritima* is a more extended structure, or more open, than that of *S. enterica* (Fig. 2)

### Structural similarity of domain D2 to antifreeze protein

Antifreeze protein Type III (PDB-id: 6AME) has been identified as the closest structural homolog to the D2 domain with an RMSD of 1.7 Å over 60 aligned residues, and a sequence identity of 15% with the highest Z-score of 8.7. The structure of the C-terminal antifreeze-like (AFL) domain of human sialic acid synthase (SAS, PDB-id: 1WVO) also showed structural similarity to the D2 domain of FlgA with an RMSD of 1.5 Å over 61 aligned residues and a sequence identity of 15% with the highest Z-score of 8.3^20^. The AFL domain in human SAS seems to play an important role in substrate binding^21^,^22^. In spite of the rather poor alignment statistic, the topology of both structures is very similar (Supplementary Fig. S3).

### Role of Domain D3 in FlgA Function

**Figure 3:**
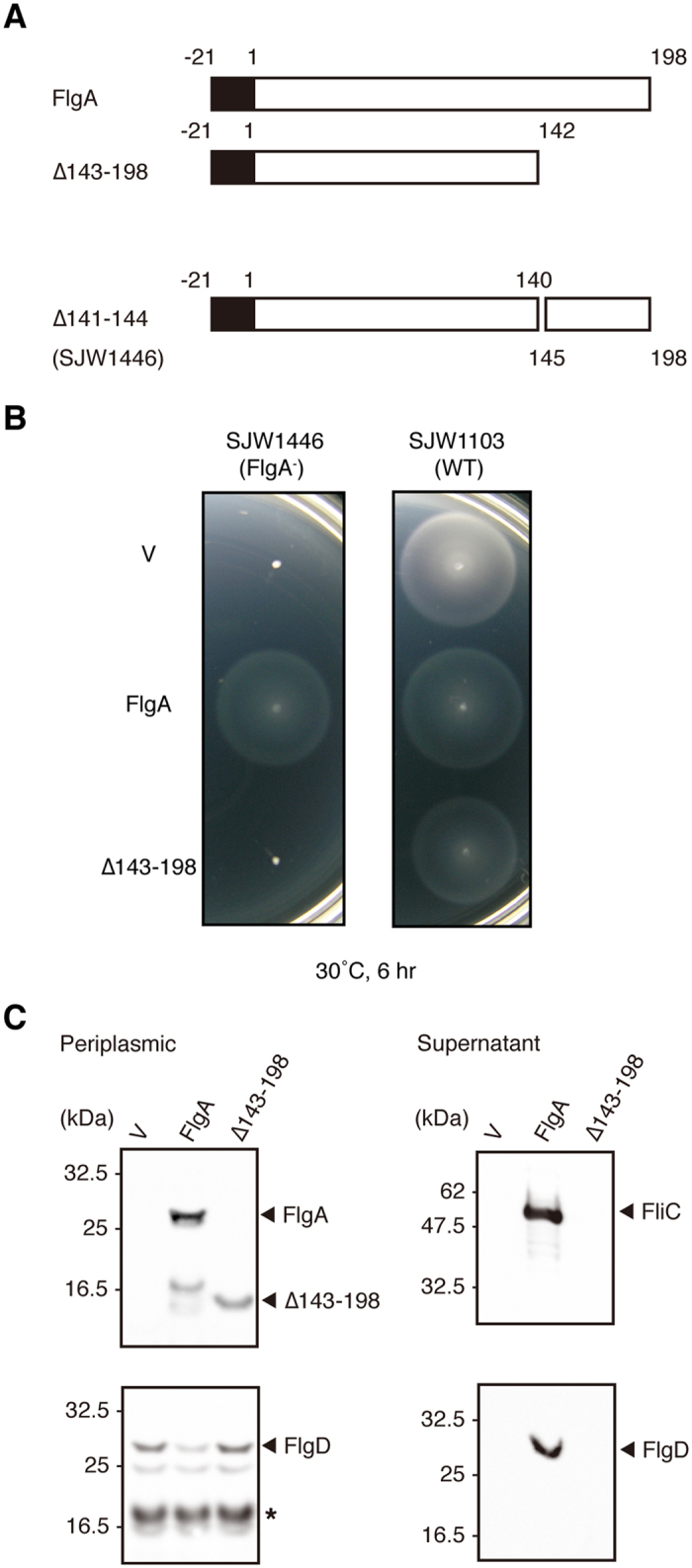
Domain organization of *S. enterica* FlgA. (**A**) Domain structures of FlgA used in this experiment. (**B**) Swarming motility of and protein secretion by wild-type (SJW1103, WT) and the *flgA* deficient strain (SJW1446, FlgA^−^) with an empty vector (V) or harboring a plasmid expressing either FlgA or FlgA(∆143–198). Swarming motility on soft agar plates. Complementation of the *flgA* deficient strain SJW1446 with *trc*-based plasmids encoding either FlgA or FlgA(∆143–198) (*left*) and the negative dominant effect on wild-type motility of SJW1103 (*right*). (**C**) Secretion assay by fractionating proteins (the periplasmic fraction *on the left* and the supernatant fraction *on the right*). Protein bands are visualized using antibodies against *S. enterica* FlgA, FlgD, and FliC. A degradation product of FlgD found in the periplasmic fraction is marked with an asterisk. Molecular marker positions are indicated *on the left*.

*S. enterica* strain SJW1446, which is non-flagellated due to a defect in the *flgA* gene^14^ that results in deletion of residues V141-G144 (∆VKAG), was used as a host cell for genetics. To examine the role of D3, we constructed a deletion variant of FlgA, FlgA_∆143–198_, lacking residues 143–198 that form domain D3 (Fig. 3A). This deletion variant neither complemented the *flgA* (∆141–144) allele of SJW1446, nor had any effect on motility of the wild-type strain SJW1103 (Fig. 3B). However, FlgA_∆143–198_ was secreted properly into the periplasm as well as wild-type FlgA (Fig. 3C). Degradation products of FlgA were also detected around the molecular size of FlgA_∆143–198_. In the periplasm, FlgA might be susceptible to proteolytic digestion. The hook-cap protein, FlgD, accumulated in the periplasm of both SJW1446 harboring the empty vector and the mutant strain producing FlgA_∆143–198_ (Fig. 3C), indicating the failure of LP-ring assembly. Neither FlgD nor FliC was found in the culture supernatant from cells harboring FlgA_∆143–198_ (Fig. 3C). FlgA_∆143–198_ did not exert a significant negative dominant effect on motility of SJW1103 (which also possesses a copy of the wild-type gene) suggesting that it failed to bind FlgI due to its lack of the D3 domain (Fig. 3B).

### Evidence of the Functional Relevance of Both Conformations

**Figure 4 f4:**
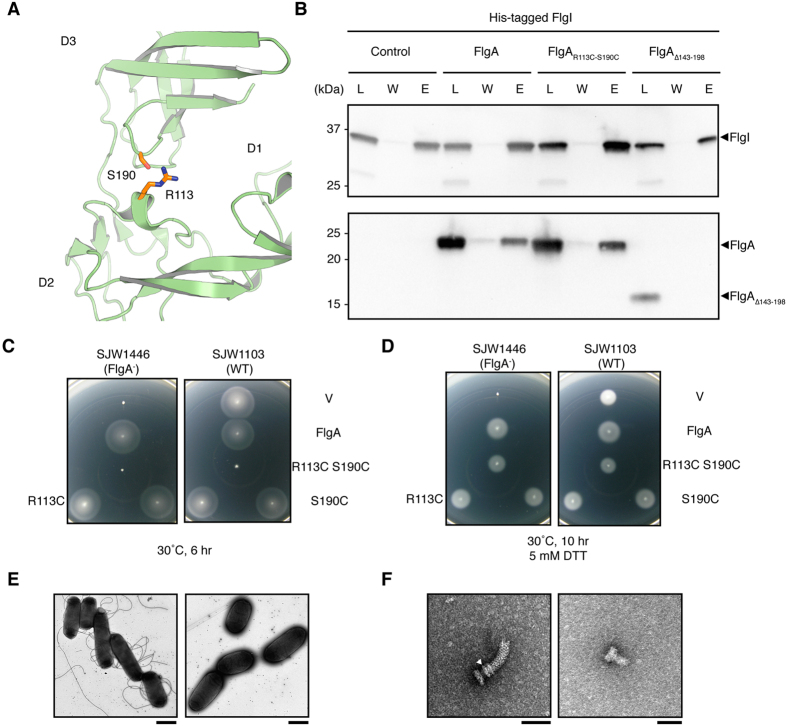
Effects of an engineered disulfide bond on the chaperone function. Cysteine substitution creates a novel disulfide bond that mimics the closed conformation of FlgA and eliminates motility. (**A**) Residues R113 and S190 shown in *stick* representation in the closed structure of FlgA. Cα atoms of R113 and S190 are 6.3 Å apart in the closed form and 17.5 Å apart in the open form. (**B**) Immunoblot showing the result of the pulldown assay of FlgA, FlgA_R113C-S190C_ and FlgA(∆143–198) with His-FlgI as a probe. Load (L), wash (W), and elution (E) are shown. Swarming motility assay on tryptone agar plates for complementation of SJW1446 (FlgA^−^) and negative dominance effect on SJW1103 (WT) transformed with a plasmid expressing the disulfide bond-locked closed form of FlgA. Assays were carried out in the absence (**C**) and presence (**D**) of 5 mM DTT. (**E**) Electron micrographs of cells of HK1431 (*left*) and HK1500 (*right*). Scale bars are 1 μm. (**F**) An isolated hook-basal body from HK1431 (*left*) and a basal body from HK1500 (*right*). Scale bars are 50 μm. The P-ring in the basal body is indicated by a *white arrowhead*.

By studying the 3D structures of FlgA, we identified a pair of residues, R113 and S190, which are within a suitable distance for cross-linking with a disulfide bond that keeps the structure of FlgA in the closed conformation (Fig. 4A). To understand how the conformational difference between the open and closed forms of FlgA is related to its function, a disulfide bond was engineered by replacing these residues with cysteines (R113C and S190C, respectively). The disulfide bond forces FlgA to stay in the closed form and constrains the movement of domain D3. Pull-down experiments revealed that FlgA and FlgA_R113C-S190C_ both bound to His-FlgI; however, FlgA_∆143–198_ could not, suggesting that the C-terminal domain of FlgA is involved in binding to FlgI and that this domain is still intact for binding of FlgA_R113C-S190C_ (Fig. 4B). The complete loss of motility in SJW1446 complemented with the plasmid expressing FlgA_∆143–198_, is explained by the lack of binding to FlgI (Fig. 3B). Before testing the effect of this double-mutation, the complementation of FlgA with each of the single mutations R113C and S190C on the motility of SJW1446 was tested (Fig. 4C). While each of these single-substitution mutants rescued the functional flagellar assembly of SJW1446, FlgA_R113C-S190C_ strongly suppressed the motility of SJW1103, presumably by blocking flagellar assembly (Fig. 4C). In the presence of dithiothreitol (DTT), which reduces disulfide bonds, the double mutant FlgA_R113C-S190C_ was able to rescue the motility of SJW1446 and had no effect on the motility of SJW1103 (Fig. 4D). The wild-type strain complemented with pHMK339 (FlgA, hereafter called HK1431) produced flagellar filaments, but in contrast with pHMK788 (FlgA_R113C-S190C_, hereafter called HK1500), no flagellar filament was produced (Fig. 4E). Isolated hook basal-bodies from these strains were morphologically different. A rivet structure, which lacks the LP-ring of the hook basal-body, was isolated from the wild-type strain expressing FlgA_R113C-S190C_ (Fig. 4F). These results indicate that the closed form of FlgA can bind to FlgI, but cannot proceed with subsequent steps, thereby inhibiting FlgI from forming the P-ring.

### Structural flexibility of FlgA

In solution, both FlgA and FlgA_R113C-S190C_ existed as monomers exclusively (Supplementary Fig. S5). Disulfide bond formation in FlgA_R113C-S190C_ was suggested by gel-shift assay and size-exclusion chromatography. In SDS-PAGE, FlgA_R113C-S190C_ migrated slightly more than FlgA in the absence of the reducing reagent, DTT, indicating that it folded into a more compact structure than FlgA (Supplementary Fig. S6). In addition, the elution of FlgA_R113C-S190C_ by size-exclusion chromatography was more retarded than that of FlgA, indicating a more compact molecular shape, and normal mobility was restored in the presence of DTT (Supplementary Fig. S6).

**Figure 5:**
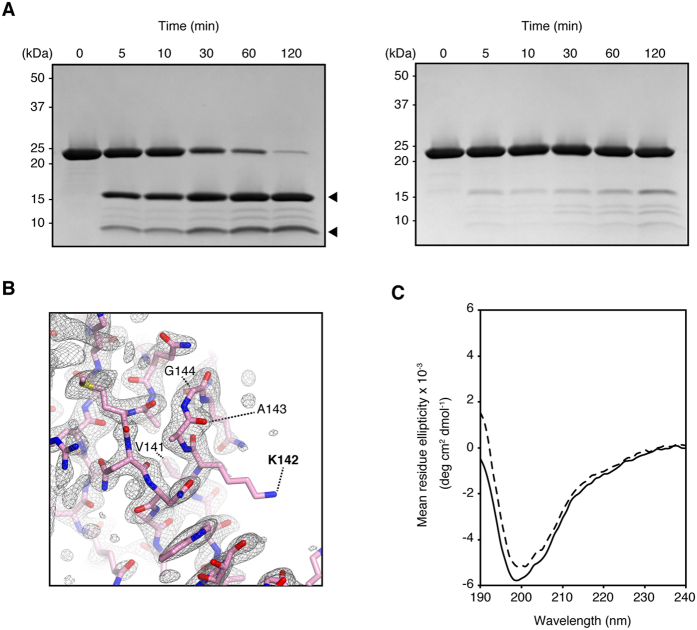
Analysis of protein stability by proteolytic digestion. Limited proteolysis of FlgA (*left*) and FlgA_R113C-S190C_ (*right*) with Lys-C endoprotease (**A**). A focused view around residues V141–G144 that are lacking in FlgA of SJW1446. A Sigma-A weighted 2m*F*o-D*F*c map generated by phenix_composite_map was shown as *mesh* by PyMOL with contour level of 1.0. The open form of FlgA was shown as *ribbon*. These residues including the protease digestion site are labeled (**B**). CD spectra of the proteins, FlgA (*solid line*) and FlgA_R113C-S190C_ (*dashed line*), were measured (**C**) from 190 to 240 nm.

FlgA and FlgA_R113C-S190C_ were subjected to protease digestion with endoprotease Lys-C. In FlgA, major proteolytic fragments appeared shortly after the reaction commenced; however, FlgA_R113C-S190C_ was resistant to digestion (Fig. 5A). The digestion site could reside between residues K142 and A143, judging from the size of the fragments, which overlaps the *flgA* (∆141–144) allele of SJW1446 (Fig. 5B). This short segment connects domains D2 and D3 and is accessible to the solvent. The strain, SJW1446, harboring pHMK714 (∆141–144), was not motile even upon overexpression of FlgA_∆141–144_ from the plasmid (Supplementary Fig. S4). The resulting flagellar growth defect was ascribed to the loss of this short segment. FlgA_∆141–144_ could be quite unstable so that it is not detected in the periplasm (Supplementary Fig. S4). Even If the deletion is acceptable to the protein, the loss of this linker might restrict the movement of D3 relative to D1, thereby suppressing the chaperone function of FlgA for FlgI. In solution, FlgA and FlgA_R113C-S190C_ showed similar CD spectra (Fig. 5C). Calculated secondary structure contents of α and β structures were 19% and 24% for FlgA, and 19% and 27% for FlgA_R113C-S190C_, respectively. No significant secondary structure transition was found in both proteins. A structural difference between FlgA and FlgA_R113C-S190C_ is solely limited to the D3 positions in the structures.

### Quantitative analysis of the interaction of FlgA with its cognate partner, FlgI

The binding of both FlgA and FlgA_R113C-S190C_ to their substrate, FlgI, was investigated using Surface Plasmon Resonance (SPR). Kinetic constants for FlgA and FlgA_R113C-S190C_ against immobilized FlgI, a ligand on the CM5 chip (GE Healthcare), were measured by assuming a two-state reaction model, allowing for conformational changes after complex formation (Supplementary Fig. S7), according to the manufacturer’s protocol (Table 2). Affinity constants (*K*_D_) of FlgA and FlgA_R113C-S190C_ against the ligand, FlgI, were estimated as almost identical, indicating no significant difference in binding FlgI. On the other hand, we analyzed the binding to their substrate, FlgI, by immobilizing either FlgA or FlgA_R113C-S190C_ to the CM5 chip (Supplementary Fig. S7). In this case, the *K*_D_ of FlgA increased to over twice that of FlgA_R113C-S190C_, indicating that the binding of FlgA_R113C-S190C_ to FlgI is slightly stronger than that of FlgA.

## Discussion

The present X-ray structural analysis of FlgA, the flagellar P-ring chaperone, showed unique structural features with no structural similarity to other flagellar chaperone proteins that function in the cytoplasm^11^,^23^. Here, structural snapshots of FlgA, revealed by X-ray crystallographic studies, suggest that the conformational difference between the two structures of the chaperone may help to explain flagellar P-ring formation (Supplementary Movie S1). Based upon the structural difference induced by crystal packing, we tested whether the closed and open forms were possible in solution. Functional assays exploring the structural difference between FlgA and FlgA_R113C-S190C_ suggested a difference in binding to FlgI. They also showed different phenotypes in *S. enterica* strains for flagellar P-ring formation; therefore, structural flexibility of the protein is essential and FlgA is able to bind to FlgI via its C-terminal domain (Figs 3, [Fig f4] and 4). FlgA_R113C-S190C_ is locked in one conformation, while FlgA adopts intermediate structures upon immobilization, suggesting that FlgA might exist in the periplasm as a flexible intermediate structure, or in equilibrium between the open and closed forms defined here.

The closed form of FlgA is reminiscent of the PapD structure, an immunoglobulin-like periplasmic chaperone for P pilus biogenesis^24^,^25^. In the PapD structure, a deep cleft between domains constitutes the substrate recognition site; however, the cleft between FlgA domains D1 and D3 in the closed form might not be suitable for FlgI binding because the C-terminal domains of both forms of FlgA showed similar affinity for FlgI binding. Upon binding to FlgI, FlgA may adopt the closed structure, since FlgA_R113C-S190C_ showed a strong negative dominant effect on motility of the wild-type strain (Fig. 3). Although a small difference at μM order in the *K*_D_ values could not be fully explained, the negative dominance effect of FlgA_R113C-S190C_, it might be effective for P-ring assembly. The *K*_D_ values are not small enough for simple complex formation (1:1 binding), but might be reasonable for the energy-independent translocation of FlgI in the periplasm. FlgI is released into the periplasm right after secretion by way of the general secretion pathway, and it should be transported to nascent flagellar structures by an unknown sorting mechanism. If FlgA is a pilot for FlgI after complex formation, promoting its localization at the destination, perhaps FlgA appears in the periplasm in the closed form prior to FlgI translocation and then binds to FlgI to recruit it to the flagellar basal body, preventing premature formation of the P-ring.

In case of a pilotin-secretin complex in the Type III secretion system of *Shigella flexneri*, MxiM and MxiD were intensively studied by X-ray crystallography and nuclear magnetic resonance spectroscopy. Pilotin MxiM binds to the C-terminus of the secretin MxiD to allow proper translocation to the outer membrane in the periplasm with structural rearrangement of secretion^26^,^27^. If the C-terminal domain of FlgA binds to FlgI, the rest of the protein would be important for translocation to the periplasm. Domain D2 may target PG through the β-clip fold. Structural similarity of domain D2 to proteins with β-clip folds supports the idea that binding to the PG layer occurs through the hydrophilic surface of domain D2^28^. This hypothesis implies that the role of FlgA in P-ring formation is to assemble FlgI molecules around the nascent rod structure close to the PG layer, probably to the proximal part of the distal rod, composed of FlgG^17^. It is unknown how FlgA releases bound FlgI prior to P-ring formation. Perhaps FlgA switches conformations from the closed to the open form after interacting with cellular components or other flagellar proteins.

Finally, crystal structures of FlgA will allow future mutagenesis studies to better define its role in FlgI stabilization, polymerization, and localization, as a molecular chaperone in flagellar P-ring formation. In most Gram-negative bacteria, flagellation of cells cannot occur in the absence of FlgA. Thus, this molecule might be a target for novel antimicrobial drugs to inhibit cell flagellation and motility. Further structural information about the FlgA-FlgI complex would be advantageous in the pursuit of new therapeutic drugs.

Our results suggest a stepwise mechanism of P-ring formation mediated by the chaperone. L-ring formation in the outer membrane begins right after P-ring formation. Without a canonical PG binding region, FlgI cannot simply diffuse into the periplasm after secretion by the *Sec* system, but is probably ushered by FlgA to its destination. In addition to binding FlgI, by preventing inappropriate oligomerization, FlgA might communicate with other flagellar components to determine an exact timing and place for P-ring formation. Further structural approaches could be applied to reveal dynamics subsequent to FlgA-FlgI complex formation.

## Experimental Procedures

### Bacterial Strains, Plasmids

Bacterial strains and plasmids used in this study are detailed in Supplementary Table S1. DNA manipulations were done according to standard protocols described by Sambrook and Russell (2001). Ampicillin and chloramphenicol were added to final concentrations of 100 and 50 μg ml^−1^, respectively.

### Purification and crystallization of FlgA

Details of expression, purification, and crystallization of *S. enterica* FlgA have been described in a crystallization note^18^.

### Structure determination of FlgA

X-ray diffraction experiments were performed at beamline BL41XU of Spring-8 with approval of the Japan Synchrotron Radiation Research Institute (JASRI) (proposal no. 2007B1500). Data were indexed and integrated with MOSFLM^29^ and scaled with SCALA^30^. Details have been previously described^18^. In brief, native FlgA crystallized in space group C222 for the open form and in space group P2_1_ for the closed form (Supplementary Table S2). To solve the structure of the open form, a multi-wavelength anomalous dispersion (MAD) experiment of platinum-derivative crystals of FlgA was carried out at 3.0-Å resolution. The initial phase was calculated with SOLVE^31^ and electron density was modified with DM^32^ using native data collected at 1.95-Å resolution. Automatic model building was carried out using Arp/Warp^33^. Iterative model building and refinement were performed with COOT^34^ and Refmac^35^. Thereafter, final refinement was accomplished with Buster^36^. During refinement, manual modification was carried out using “omit map”^37^. To solve the closed form, a single-wavelength anomalous dispersion (SAD) experiment with selenomethionine-substituted FlgA (SeMet-FlgA) belonging to space group C222_1_ was carried out at 2.7-Å resolution. The native structure of the closed form crystallized in space group P2_1_ was solved by molecular replacement and refined with Phenix^38^. In the open form, the C-terminally fused linker region including a hexa-histidine (His_6_) tag (15 amino acids in length) was partly visible and could be modeled; however, the His_6_ tag part was omitted from the final model due to poor electron density. For clarity, the C-terminal linker fused to FlgA was omitted from all figures. Refinement statistics are summarized in Table 1. A search for structurally homologous proteins in the Protein Data Bank (PDB) was performed with DALI^20^.

### Motility assay and protein secretion experiments

The *S. enterica flgA* mutant strain SJW1446 was used as the host for *trc*-based plasmids for complementation assays on semi-solid agar plates (Table S1). The *S. enterica* wild-type strain SJW1103 was used as the host for the negative dominance assay as well. Assays were performed at 30 °C. Periplasmic fractions were prepared as described previously^39^. Secreted proteins were detected by Western blotting. Fractionated proteins were subjected to SDS-PAGE and then electroblotted to nitrocellulose membranes, where they were visualized using a photoimager LAS3000 (Fuji film, Tokyo, Japan) or an imaging system ChemiDoc XRS^+^ (Bio-Rad, Hercules, CA, USA) with antibodies against *S. enterica* FlgA, FlgD, and FliC (MBL, Nagoya, Japan).

### DNA sequencing

Genomic DNA was isolated from *S. enterica* SJW1446^13^ using Gentra Puregene Yeast/Bacteria kit (QIAGEN, Gaithersburg, MD). The *flgA* gene was amplified by PCR using genomic DNA as a template. The gene was then cloned into the *Nde*I/*Bam*HI site in pHMK11 to create pHMK714. The *flgA* DNA sequence was confirmed with an ABI3700 using a BigDye terminator cycle sequencing kit (Applied Biosystems, Foster City, CA).

### Pull-down Assays

Pull-down assays of FlgA-FlgI complex formation with TALON metal affinity chromatography were carried out following the manufacturer’s instructions (Clontech, Mountain View, CA). Cell extracts prepared from *E. coli* Origami2 (DE3), harboring the N-terminally His-tagged FlgI (His-FlgI, pHMK385) co-expressed without FlgA (pHMK14) or FlgA proteins (pHMK720, pHMK844 or pHMK869) were loaded onto a TALON spin column. Following extensive washing with a buffer containing 10 mM imidazole, bound proteins were eluted with 300 mM imidazole and separated by SDS-PAGE, transferred to a PVDF membrane, and probed with custom antibodies raised against FlgA and FlgI (MBL, Nagoya, Japan). Membrane images were acquired with a ChemiDoc XRS^+^ system (Bio-Rad, Hercules, CA).

### Transmission electron microscopy (TEM) observation

Cells were cultured until late log phase in LB medium supplemented with Amp at 30 °C and spun down to remove culture media. After washing with phosphate-buffered saline (pH 7.4), cells were re-suspended in the buffer and applied to freshly glow-discharged carbon grids for negative staining with 1% (w/v) uranyl acetate. Images were collected on a JEM-1230R (Jeol, Tokyo, Japan) operated at 100 keV, equipped with a 2k × 2k Gatan slow-scan, charge-coupled device camera and processed with Digital Micrograph 3.1 (Gatan, Pleasanton, CA). Flagellar basal bodies were isolated as described previously with several modifications^40^. Sucrose density gradient centrifugation (20–50%(w/v)) was employed instead of a CsCl gradient.

### Limited proteolysis of FlgA

Purified FlgA and FlgA_R113C-S190C_ were mixed with endoprotease Lys-C (Roche Molecular Diagnostics, Indianapolis, IN) at a weight ratio of 250:1 (protein:protease) in 10 mM HEPES (pH 7.0), 100 mM NaCl. Reaction mixtures at each time interval were stopped by mixing with SDS loading buffer and heating at 95 °C for 5 min.

### Circular dichroism (CD) spectroscopy

Protein concentrations of FlgA and FlgA_R113C-S190C_ were determined with an Ultrospec3100pro (GE Healthcare) using extinction coefficients at 280 nm of 0.903 and 0.910, respectively. CD spectra of FlgA (0.13 mg/ml) and FlgA_R113C-S190C_ (0.12 mg/ml) were measured in 10 mM sodium phosphate buffer (pH 7.0) with a J-820 spectrophotometer (Jasco, Tokyo, Japan) at wavelengths from 190 to 240 nm at 25 °C. Data were analyzed with Spectra Manager ver. 1.55 (Jasco, Tokyo, Japan). Secondary structure contents of proteins were estimated with *K2D3*^41^.

### Surface Plasmon Resonance (SPR) analysis

All SPR experiments were carried out using a Biacore T200 (GE healthcare) at 25 °C. Proteins (His-FlgI, FlgA, FlgA_R113C-S190C_ were purified as described above. For FlgA and FlgA_R113C-S190C_, the N-terminal-fused histidine tags were removed with thrombin (GE Healthcare) prior to gel filtration. Ligand proteins were immobilized on CM5 sensor chips followed by the manufacture’s standard amine coupling protocol. Analytes were diluted with a running buffer [10 mM HEPES, 150 mM NaCl, 0.05% (v/v) Surfactant P20, (pH 7.4)]. Regeneration of the ligands was conducted by injecting 10 mM Glycine-HCl (pH 1.5). Data were processed with Biacore T200 Evaluation Software v1.0 (GE Healthcare).

Structures reported here are explained in interactive 3D at http://Proteopedia.Org/w/Samatey/2.

## Additional Information

**Accession codes:** Atomic coordinates and structure factors of the native forms (open and closed) and the SeMet-labeled form (closed) have been deposited in the Protein Data Bank (PDB; http://www.pdb.org) as accession numbers 3TEE, 3VKI and 3VJP, respectively.

**How to cite this article**: Matsunami, H. *et al*. Structural flexibility of the periplasmic protein, FlgA, regulates flagellar P-ring assembly in *Salmonella enterica*. *Sci. Rep*. **6**, 27399; doi: 10.1038/srep27399 (2016).

## Supplementary Material

Supplementary Information

Supplementary Movie S1

## Figures and Tables

**Table 1:** Refinement statistics for the open and closed forms of *S. enterica* FlgA.

	Open	Closed	Closed
	Native	SeMet	Native
Space group	*C*222	*C*222_1_	*P*2_1_
Unit-cell parameters (Å,°)	*a* = 107.52,	*a* = 53.17,	*a* = 53.93,
	*b* = 131.77,	*b* = 162.50,	*b* = 103.32,
	*c* = 49.36	c = 103.49	*c* = 85.50,
			β = 107.26
Number of molecule in the asymmetric unit	1	2	4
Resolution (Å)	19.8–1.95	24.5–2.70	25.0–2.30
*R*_work_	20.4	24.4	25.1
*R*_free_	23.4	27.7	28.1
Number atoms			
Protein	1497	2990	5980
Water	165	45	186
Ligands	5 (Glycerol) 1 (Chloride ion)	–	–
B-factors			
Protein	35.9	74.0	60.5
Water	44.7	51.3	43.8
Ligands	73.5 (Glycerol) 57.1(Chloride ion)	–	–
RMSD			
Bond length (Å)	0.015	0.004	0.004
Bond angles (˚)	1.496	1.044	0.969
Ramachandran plot (%)			
Favored	99.50	96.94	95.66
Allowed	0.50	3.06	4.34
Outliers	0.00	0.00	0.00
PDB-id	3TEE	3VJP	3VKI

Values in parentheses are for the highest resolution shell.

**Table 2:** Kinetic parameters determined by SPR experiments.

Ligand-Analyte	*k*_a1_(M^−1^s^−1^) [10^5^]	*k*_d1_(s^−1^) [10^−3^]	*k*_a2_(s^−1^)[10^−3^]	*k*_d2_(s^−1^)[10^−3^]	*K*_D_ (M) [10^−6^]
FlgI-FlgA	3.41	5.89	1.24	3.31	0.126
FlgI-FlgA_R113C-S190C_	3.35	5.16	2.17	4.72	0.106
FlgA-FlgI	0.174	9.76	0.985	1.73	0.354
FlgA_R113C-S190C_ -FlgI	0.152	5.02	0.673	0.980	0.166
