# Supplementary material for: Structural flexibility of the periplasmic protein, FlgA, regulates flagellar P-ring assembly in Salmonella enterica
Source: Sci Rep. 2016 Jun 7;6:27399. doi: 10.1038/srep27399 (PMC4895218; doi:10.1038/srep27399)
Supplement: Supplementary Information [file srep27399-s1.pdf]

Supplementary Information

Structural flexibility of the periplasmic protein, FlgA, regulates the bacterial flagellar P-ring assembly in *Salmonella enterica*.

Hideyuki Matsunami[1], Young-Ho Yoon[1], Vladimir A. Meshcheryakov[1], Keiichi Namba[2,3,4] and Fadel A. Samatey[1,*]

[1]Trans-Membrane Trafficking Unit, Okinawa Institute of Science and Technology, 1919-1 Tancha, Onna, Kunigami, Okinawa 904-0945, Japan

[2]Dynamic NanoMachine Project, International Cooperative Research Project, Japan Science and Technology Agency, 1-3 Yamadaoka, Suita, Osaka 565-0871, Japan

[3]Graduate School of Frontier Biosciences, Osaka University, 1-3 Yamadaoka, Suita, Osaka 565-0871, Japan

[4]Riken Quantitative Biology Center, 1-3 Yamadaoka, Suita, Osaka 565-0871, Japan

* Correspondence to: bakary.macy.samatey@gmail.com

Supplementary Table S1.

Strains and plasmids used in this study.

Strains or Plasmids	Genotype or description	Reference or source
Strains		
Salmonella enterica serovar Typhimurium		
SJW1103	wild-type for motility and chemotaxis	42
SJW1446	*flgA* (ΔV141-G144)	14
Escherichia coli		
DH5α	recipient for cloning	43
BL21(DE3)	*T7* expression host	Novagen
B834(DE3)	*T7* expression host	Novagen
Origami2(DE3)	*T7* expression host	Novagen
Plasmids		
pET3c	*T7* expression vector, Ap^r	Novagen
pET15b	*T7* expression vector, Ap^r	Novagen
pET22b	*T7* expression vector, Ap^r	Novagen
pHMK338	ss+FlgA-His_6 in pET22b	This study
pHMK11	modified pTrc99A vector, Ap^r	44
pHMK338	ss+FlgA-His_6 in pET22b	This study
pHMK339	ss+FlgA in pHMK11	This study
pHMK479	ss+FlgA[C36A] in pHMK11	This study
pHMK480	ss+FlgA[C59A] in pHMK11	This study
pHMK481	ss+FlgA[C36A, C59A] in pHMK11	This study
pHMK714	ss+FlgA(Δ141-144) in pHMK11	This study
pHMK748	ss+FlgA(Δ143-198) in pHMK11	This study
pHMK784	ss+FlgA[R113C] in pHMK11	This study
pHMK786	ss+FlgA[S190C] in pHMK11	This study
pHMK788	ss+FlgA[R113C, S190C] in pHMK11	This study
pACYC184	cloning vector, Cm^r and Tet^r	New England BioLabs
pHMK14	The *Cla*I-*Sal*I fragment of pET3c in pACYC184	This study
pHMK385	His_6-FlgI in pET15b	This study
pHMK720	FlgA in pHMK14	This study
pHMK841	*pelB*ss-His_6-FlgA in pET22b	This study
pHMK842	*pelB*ss-His_6-FlgA[R113C, S190C] in pET22b	This study
pHMK844	FlgA[R113C, S190C] in pHMK14	This study
pHMK869	FlgA(Δ143-198) in pHMK14	This study

The prefix “ss” denotes the original signal peptide for secretion.

Supplementary Table S2.

Summary of the data collection statistic for the closed form of FlgA crystal.

	Closed
	Native
Space group	$P2_1$
Unit-cell parameters (Å, °)	*a* = 53.93,<br>*b* = 103.32,<br>*c* = 85.50,<br>β = 107.26
Wavelength (Å)	1.0000
Resolution (Å)	25.0-2.30 (2.42-2.30)
Unique Reflections	148572 (18945)
Completeness	99.2 (96.4)
Redundancy	3.8 (3.2)
$I/\sigma(I)$	13.5 (4.2)
R_{merge}[†] (%)	7.3 (32.7)

[†]$R_{merge} = \Sigma_{hkl}\Sigma_i|I_i(hkl) - \langle I(hkl)\rangle|/\Sigma_{hkl}\Sigma_i I_i(hkl)$, where I_i(hkl) is the ith observation of reflection hkl and $\langle I(hkl)\rangle$ is the weighted average intensity for all observations i of reflection hkl. Values in parentheses are for the highest resolution shell.

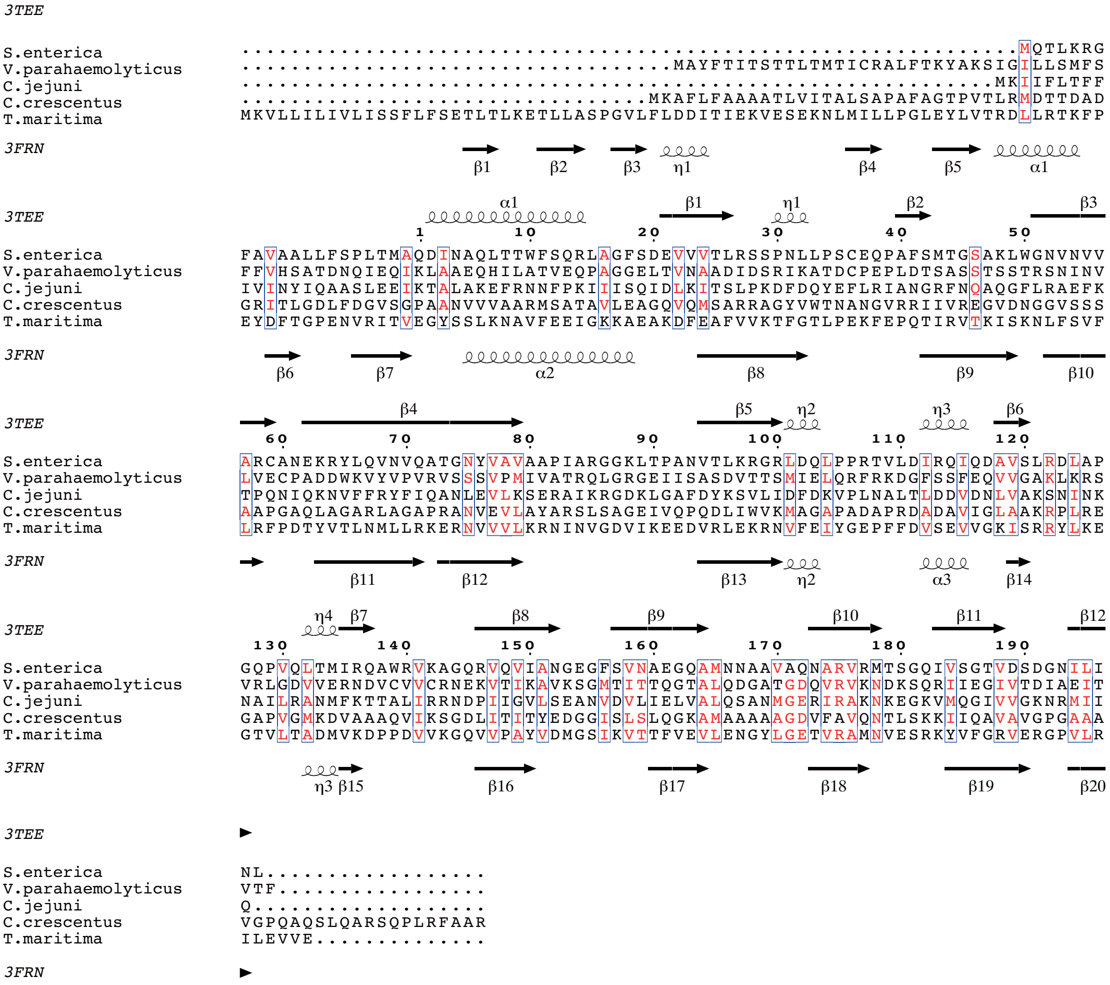

Figure S1. Sequence alignment of FlgA proteins from various organisms (UniProt; http://www.uniprot.org, P40131, Q9X9K4, Q5HV23, B8GXB9 and Q9X1M7) prepared with ESPript 3.0 (http://espript.ibcp.fr/ESPript/ESPript/). Secondary structure from *Salmonella enterica* serovar Typhimurium FlgA (PDB-id: 3TEE) depicted at the *top* and *Thermotoga maritima* FlgA (PDB-id: 3FRN) at the *bottom*. Numbering above the sequences starts from the first glutamine residue in the mature form of *S. enterica* FlgA. Similar residues are boxed and shown in red.

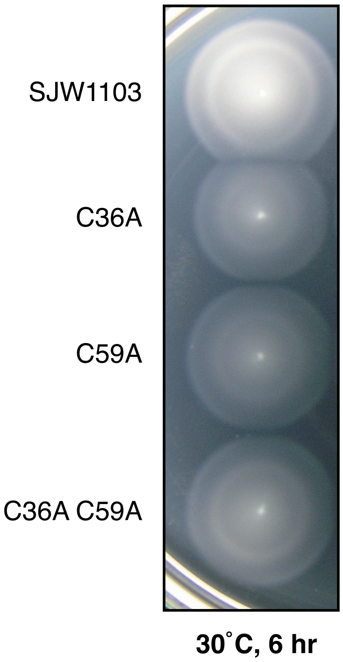

Figure S2. Swarming motility assay on soft agar plates. Complementation of FlgA variants with alanine-substitution of two cysteine residues (C36, C59 or both) in SJW1446. All alanine-mutant genes showed motile phenotypes in SJW1446 as well as SJW1103.

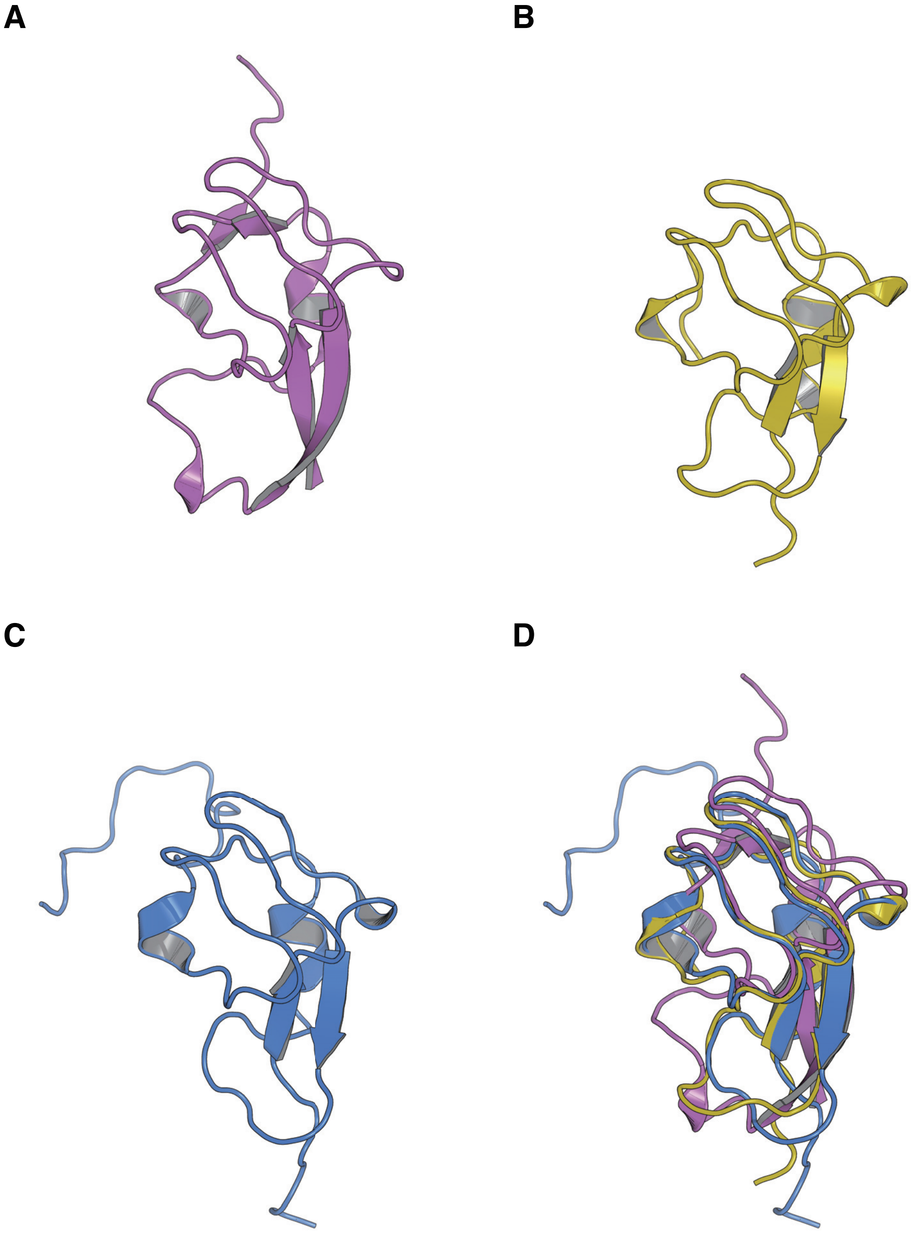

Figure S3. Cartoon representation of the structures of domain D2 of FlgA (PDB-id: 3TEE) colored in magenta (**A**), antifreeze protein Type III of ocean pout (PDB-id: 6AME) in yellow (**B**) and the C-terminal antifreeze-like domain of human sialic acid synthase (PDB-id: 1WVO) in light-blue (**C**) viewed from the β-clip fold. Superimposition of all three structures (**D**).

A

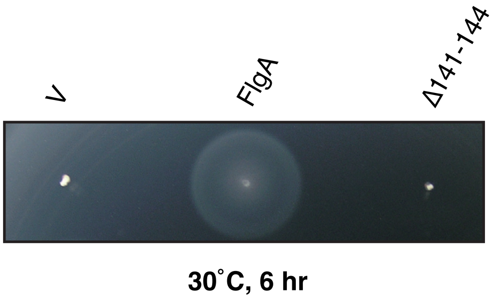

B

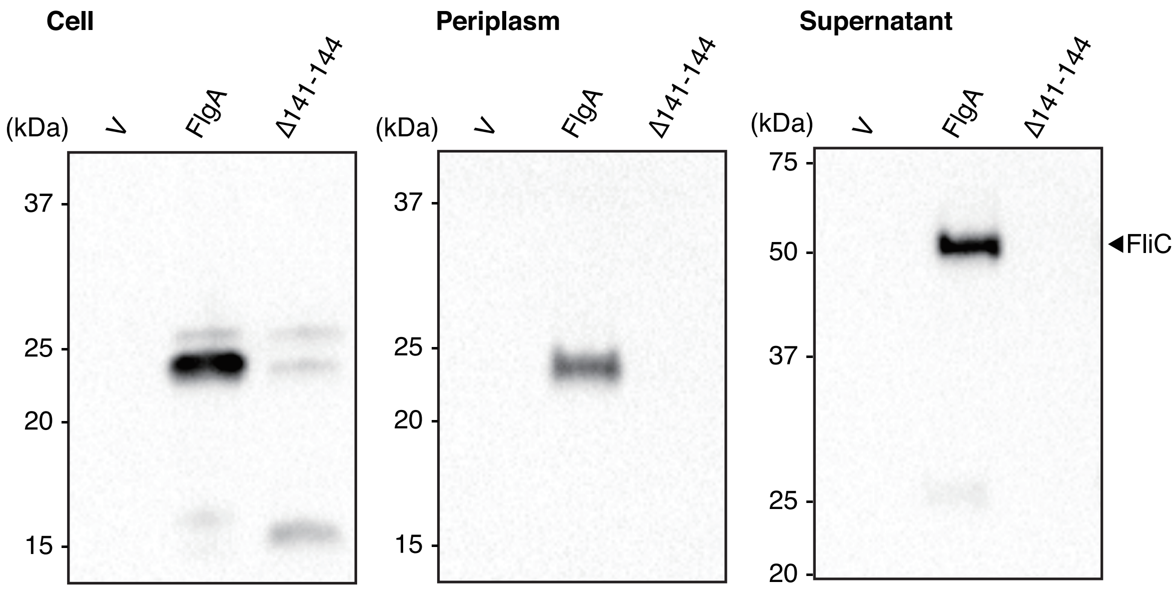

Figure S4. Characterization of FlgA (Δ141-144) from SJW1446. Swarming motility assay of SJW1446 harboring an empty vector pHMK11 (V), pHMK339 (FlgA), or pHMK714 (Δ141-144) on a soft agar plate (0.35%) (**A**). Protein expression levels of FlgA and FlgA(Δ141-144) in the cell and the periplasm fractions and FliC secretion levels in the supernatants were detected using western-blotting with antibodies against FlgA and FliC, respectively (**B**).

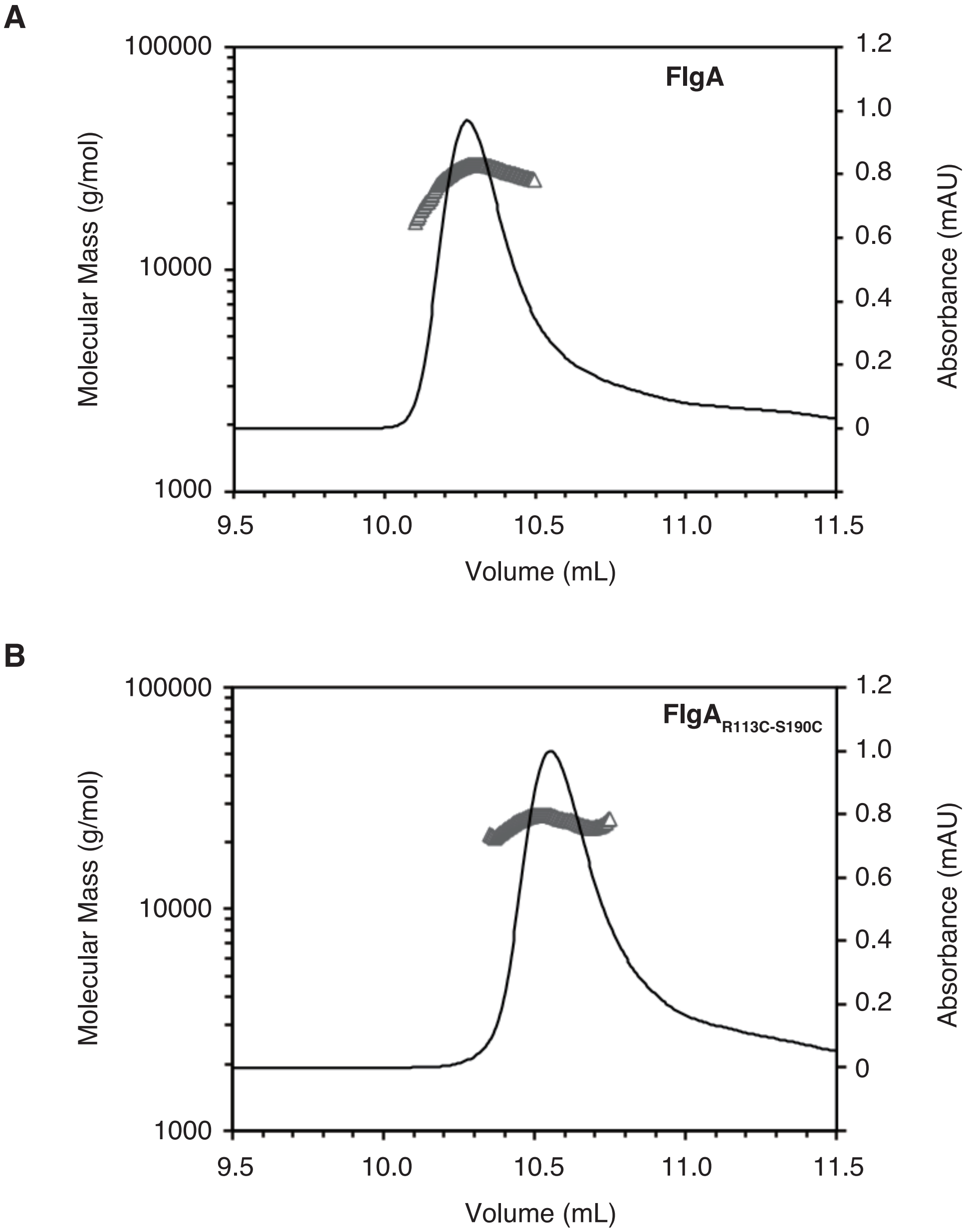

Figure S5. Size-exclusion chromatography with multi-angle light scattering (SEC-MALS). SEC-MALS experiments were carried out on a Waters Alliance e2695 Separations Module HPLC system (Waters Corporation, Milford, MA) equipped with a DAWN HELIOS 8+ MALS detector (Wyatt Technology, Santa Barbara, CA), an Optilab T-rEX differential refractive index detector (Wyatt Technology, Santa Barbara, CA), and a Waters 2489 UV/Vis detector (Waters Corporation, Milford, MA)

in line. A 20 μl volume of the purified FlgA (**A**) or $FlgA_{R113C-R190C}$ (**B**) proteins (0.5 mg/mL) was injected onto a silicone size exclusion column WTC030S5 (Wyatt Technology, Santa Barbara, CA) in phosphate-buffered saline (pH 7.3) at a flow rate of 0.4 mL/min. Data were analyzed using a software package ASTRA ver. 6.1.2.84 (Wyatt Technology, Santa Barbara, CA). UV and LS were shown as solid and dashed lines, respectively. The molecular mass distributions of FlgA and $FlgA_{R113C-S190C}$ are shown as *triangles*.

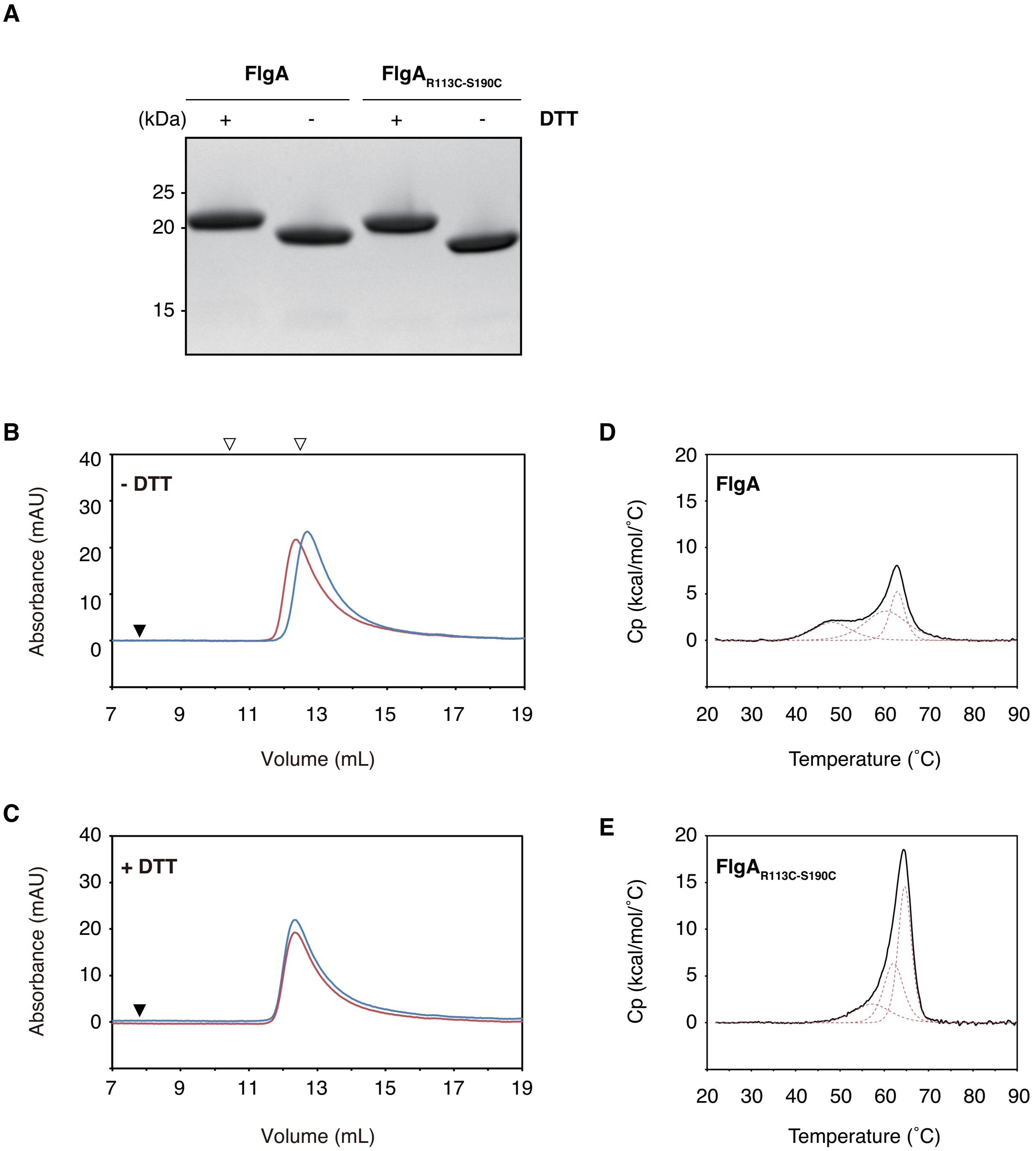

Figure S6. Evaluation of the disulfide bond formation in $FlgA_{R113C\text{-}S190C}$. Proteins (2 μg) of FlgA and $FlgA_{R113C\text{-}S190C}$ for gel-shift assay were prepared in the presence or absence of a reducing reagent dithiothreitol (DTT) and separated in a 4–20% precast polyacrylamide gel (Bio-Rad, Hercules, CA). Proteins are visualized with CBB and

digitized using the ChemiDoc XRS+ system (Bio-Rad, Hercules, CA) (**A**). Proteins of FlgA (*red solid line*) and $FlgA_{R113C-S190C}$ (*blue solid line*) (40 μg, 0.4 mg/mL) were loaded onto a Superdex 75 10/300 GL column pre-equilibrated with phosphate-buffered saline (PBS). In the absence of DTT, the peak elution volumes of FlgA and $FlgA_{R113C-S190C}$ were 12.3 mL and 12.7 mL, respectively. Gel-filtration standard proteins Ovalbumin (Mw. 44,000) and Myoglobin (Mw. 17,000) (Bio-Rad, Hercules, CA) were eluted peaking at 10.3 ml and 12.4 ml, respectively (*open triangles*). The void volume of the column (7.7 ml) was indicated as *filled triangle* (**B**). For the reducing condition, proteins were incubated in the presence of 5 mM DTT for 20 min prior to injection and subsequently separated in PBS containing 1 mM DTT. In the presence of DTT, the peak elution volumes of FlgA and $FlgA_{R113C-S190C}$ were 12.3 mL and 12.4 mL, respectively (**C**). Comparison of the thermal stability of FlgA and $FlgA_{R113C-R190C}$. Differential scanning calorimetry (DSC) measurements were carried out using a MicroCal VP-Capillary DSC system (Malvern Instruments, Malvern, UK). The heat capacity changes of FlgA (**D**) and $FlgA_{R113C-R190C}$ (**E**) in 10 mM HEPES-NaOH (pH 7), 100 mM NaCl (0.4 mg/mL) were monitored from 20 to 90˚C at a raising rate of 60˚C/hr (*black solid lines*). The curves were fitted to three-state transitions and the transition midpoint values (T*m1,* T*m2 and* T*m3*) were estimated to 60.4, 63.0 and 48.3 for FlgA, and 57.3, 62,1 and 64.6 for $FlgA_{R113C-R190C}$, respectively (*red dash lines*).

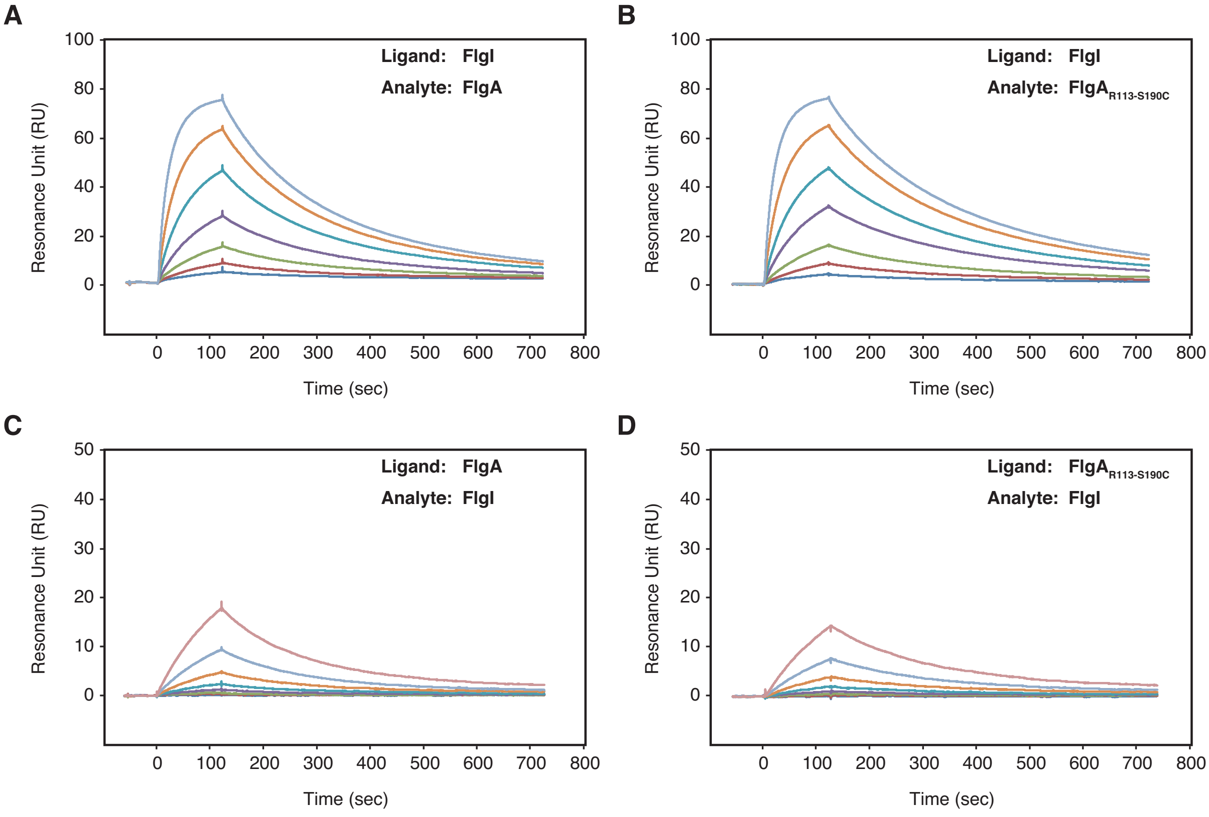

Figure S7. Protein-protein interactions were measured with surface plasmon resonance. Sensor graphs obtained by the experiments are shown. Analyte concentrations of FlgA (**A**) and FlgA$_{R113C-S190C}$ (**B**) were prepared at 2, 4, 8, 16, 32, 64, and 128 nM against the ligand, FlgI. For FlgI, analyte concentrations were prepared at 4, 8, 16, 32, 64, and 128 nM against ligands FlgA (**C**) and FlgA$_{R113C-S190C}$ (**D**).

Supplementary Movie S1.

flga_Samatey.mov

Movie S1 shows a linear interpolation morph (methods at http://proteopedia.org/w/morphs) between the open and closed forms of FlgA. The FlgA chain is represented as a backbone trace, colored blue at the amino terminus and red at the carboxyterminus, with a spectral sequence of colors in between. The movie was kindly generated by Eric Martz using Jmol (http://jmol.org) to create jpeg images that were assembled with QuickTime Pro. The wmv format was exported with Flip4Mac. Interactive 3D graphics of FlgA are explained at http://proteopedia.org/w/Samatey/2

Supplementary References:

42. Yamaguchi, S., Fujita, H., Sugata, K., Taira, T. & Iino, T. Genetic analysis of H2, the structural gene for phase-2 flagellin in Salmonella. J Gen Microbiol **130**, 255-65 (1984).

43. Taylor, R.G., Walker, D.C. & McInnes, R.R. E. coli host strains significantly affect the quality of small scale plasmid DNA preparations used for sequencing. Nucleic Acids Res **21**, 1677-8 (1993).

44. Kojima, S., Furukawa, Y., Matsunami, H., Minamino, T. & Namba, K. Characterization of the periplasmic domain of MotB and implications for its role in the stator assembly of the bacterial flagellar motor. J Bacteriol **190**, 3314-22 (2008).
